
JOURNAL INFORMATION
==============================
NLM Title Abbreviation: ScientificWorldJournal
Journal ID: ScientificWorldJournal
NLM Journal ID: 101131163
Journal ID: TSWJ

The Scientific World Journal

ISSN: 2356-6140
EISSN: 1537-744X
Publisher: Wiley

ARTICLE INFORMATION
==============================
PMCID: PMC5936489
PMID: 16075153
DOI: 10.1100/tsw.2005.66
Article ID: 976287
Article version: 1
Subjects: Research Article

Inferior Vena Cava in Urology: Importance of Developmental Abnormalities in Clinical Practice

Srivastava Aneesh 1 *
Singh Kamal Jeet 2
Suri Amit 2
Vijjan Vivek 2
Dubey Deepak 3
1 Department of Urology, Sanjay Gandhi Post Graduate Institute of Medical Sciences, Lucknow, India
2 Senior Resident, Department of Urology, Sanjay Gandhi Post Graduate Institute of Medical Sciences, Lucknow, India
3 Assistant Professor Department of Urology, Sanjay Gandhi Post Graduate Institute of Medical Sciences, Lucknow, India
*Aneesh Srivastava: anees@sgpgi.ac.in
Publication date: 2005
Electronic publication date: 2005 Jul 22
Volume: 5
First page: 558
Last page: 563
Received 2005 Mar 11; Revised 2005 Jun 12; Accepted 2005 Jun 12
Copyright: Copyright © 2005 A. Srivastava et al.
Copyright year: 2005
License: This is an open access article distributed under the Creative Commons Attribution License, which permits unrestricted use, distribution, and reproduction in any medium, provided the original work is properly cited.
License URL: https://creativecommons.org/licenses/by/3.0/

Keywords: IVC, developmental anomalies, clinical significance

==============================
Research Article
*TheScientificWorldJOURNAL* (2005) 5, 558–563
ISSN 1537-744X; DOI 10.1100/tsw.2005.66

### Inferior Vena Cava in Urology: Importance of Developmental Abnormalities in Clinical Practice

Aneesh Srivastava[1,*], Kamal Jeet Singh[2], Amit Suri[2], Vivek Vijjan[2], and Deepak Dubey[3]

*[1]Additional Professor, Department of Urology, [2]Senior Resident, Department of Urology, [3]Assistant Professor Department of Urology, Sanjay Gandhi Post Graduate Institute of Medical Sciences, Lucknow, India*

**E-mail:** anees@sgpgi.ac.in

*Received March 11, 2005; Revised June 12, 2005; Accepted June 12, 2005; Published July 22, 2005*

---

**KEYWORDS**: IVC, developmental anomalies, clinical significance

---

### INTRODUCTION

Anomalies of the inferior vena cava (IVC) have been known since 1793, when Abernethy first described a congenital, mesocaval shunt and azygous continuation of the IVC in a 10-month-old infant with polysplenia and dextrocardia[1].

The IVC is formed by a complex process of embryogenesis during the sixth to tenth week of gestation. It forms from continuous appearance and regression of the three paired veins: posterior cardinal, subcardinal, and supracardinal.

Improper completion of the developmental process may result in at least 14 anatomic anomalies, out of which the following four are usually encountered in clinical practice: duplication of the IVC, transposition or left-sided IVC, retroaortic left renal vein, and circumaortic left renal vein[2]. It is suggested that the preoperative diagnosis of the vascular anomalies reduces the complication rate of abdominal vascular procedures[3,4]. Our vast experience with approximately 400 kidney donors who were evaluated preoperatively with spiral CT scan with three-dimensional reconstruction (3D) reconfirmed this view[5]. Thereafter, it became easier to choose the side and decide between laparoscopic vs. open approach[6]. This prompted us to write the present article focusing on those developmental anomalies of the IVC that may be encountered by the urologist and their implication on the clinical practice.

### EMBRYOGENESIS OF THE IVC

During the early weeks of development, the blood from the body wall caudal to the heart is drained by posterior cardinal veins, while that from the viscera is drained via vitelline veins. Subsequently, subcardinal veins develop ventromedial to the posterior cardinal veins and ventrolateral to the aorta. The intersubcardinal anastomosis develops between paired subcardinal veins and superior mesenteric artery.

*Corresponding author. Tel: +91-522-2668678/Fax: +91-522-2668017
©2005 with author.

At the same time, there occurs anastomosis between the right subcardinal veins and hepatic segment of the IVC, which is formed from vitelline veins. Blood return from the lower extremities is shunted through postsubcardinal veins as the cranial part of the posterior cardinal veins begins to atrophy. This process establishes prerenal division of the IVC.

Next, supracardinal veins (paired) appear and lie dorsomedial to the posterior cardinal veins and dorsolateral to the aorta. Anastomosis develops between the supracardinal veins and subcardinal veins. The supracardinal system then separates into cranial (azygous) and caudal (lumbar) ends. Meanwhile, there occurs anastomosis between the two posterior cardinal veins and between the posterior and lumbar supracardinal veins. With further atrophy of the posterior cardinal veins, the blood from lower extremities is shunted through the supracardinal system to the suprasubcardinal anastomosis and then to prerenal division of the IVC. Finally, the left supracardinal vein is one of the last veins to disappear[7].

In summary, the IVC is composed of four segments (Fig. 1): hepatic, suprarenal, renal, and infrarenal. The hepatic portion is derived from vitelline veins. The right subcardinal vein develops into the suprarenal portion of the IVC, while the renal segment develops from right suprasubcardinal and postsubcardinal anastomoses. The infrarenal portion of the IVC is generally believed to arise from the right supracardinal vein, although development of this part of the IVC is controversial.

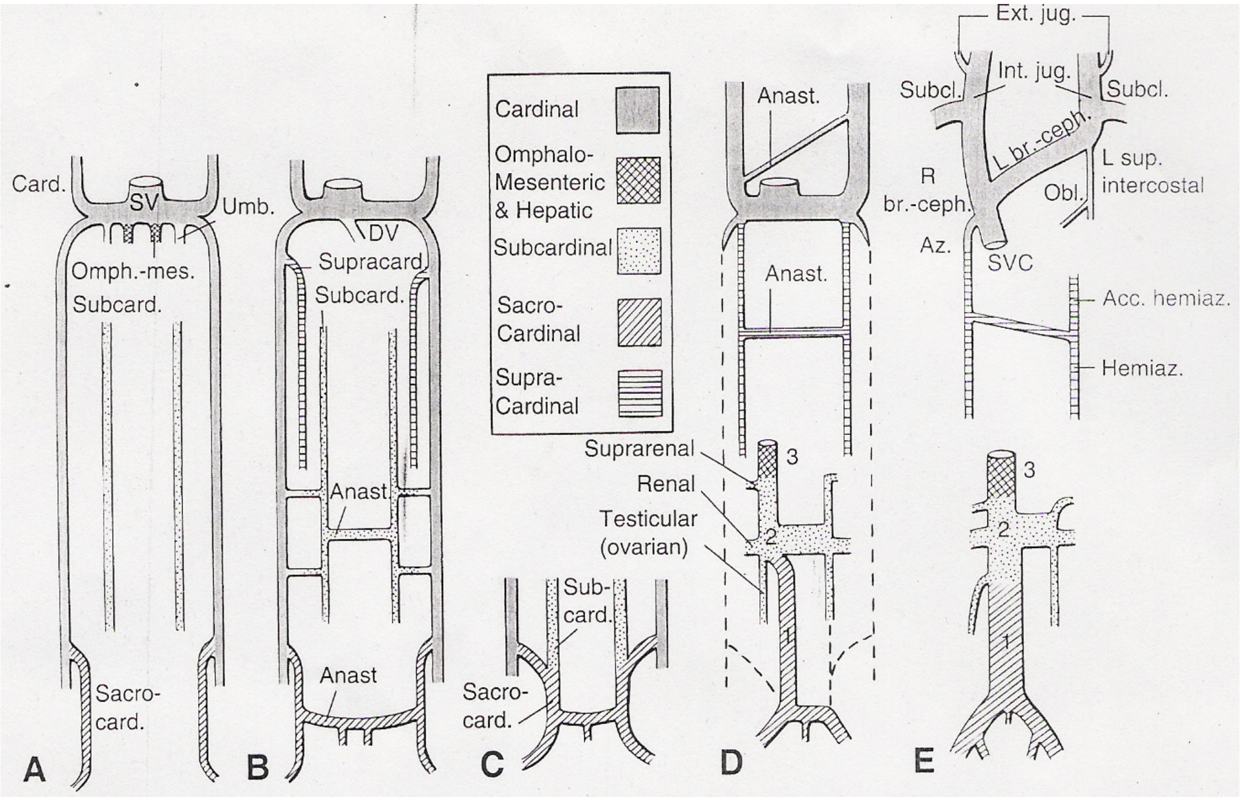

**FIGURE 1**. Contribution of various veins in successive development of the IVC. The transformations of the cardinal, subcardinal, sacrocardinal, and supracardinal systems are indicated by appropriate shading. In D and E: (1) sacrocardinal veins, (2) subcardinal veins, (3) hepatic veins.

### IMAGING MODALITIES

Several imaging techniques are now available for use in evaluation of renal vascular anatomy. They include angiography, conventional and spiral computerized scan (spiral CT), and magnetic resonance imaging (MRI).

Angiographic techniques, despite being most accurate in defining the abdominal arterial tree, are currently not in vogue because of their invasive nature and associated vascular complications. Also, angiography does not define the venous anatomy accurately. Intravenous digital subtraction angiography (DSA) entails injection of the contrast intravenously to delineate vascular anatomy. Intravenous DSA also has limitation in the form of the requirement of a high dose of contrast, poor resolution, superimposition of multiple vessels, and imaging artifacts caused by motion and bowel peristalsis. Intra-arterial DSA entails injection of contrast material through a percutaneous-introduced catheter and it provides better resolution with fewer imaging artifacts than intravenous DSA.

Spiral CT uses new techniques to view large vessels such as the aorta and its branches[8,9]. Data are acquired from multiple contiguous, 360-degree tube rotations at a table feed speed of 3–10 mm/sec during intravenous administration of contrast material. The acquired data volume is reconstructed into axial sections with 50% overlap to achieve a 3D image. Spiral CT angiography provides excellent anatomical detail of the aorta and its branches. This modality is used successfully for the screening of potential renal donors[10]. Spiral CT with 3D reconstruction has become the standard method of imaging renal donors at our institute. In 120 donors studied by this method, sensitivity and specificity in depicting arterial and venous anatomy was 91.6 and 98.2%, 96.7 and 90.0%, respectively[4].

MR angiography has rapidly become an accepted modality because there is no need for iodinated contrast and ionizing radiation. It is well suited for larger vessels including renal arteries, veins, and aorta[11,12]. Several pulse sequences are used for imaging renal arteries. The initial sequence is “black blood imaging” in which blood appears black on T1-weighed, spin echo images. The next sequence is “time of flight” in which flowing blood appears bright. The third sequence is “phase contrast imaging” and is used to image flowing blood by means of a velocity-induced phase in which the inflowing blood appears as bright as in the second sequence. Problems with MR angiography like respiratory motions, vessel overlap, and inherent complex flow pattern have been largely solved with the use of Gadolinium. Gadolinium shortens T1 relaxation time of blood to allow the imaging of vessels independent of flow. Gadolinium contrast images are similar to DSA[12]. MR angiography is not easily performed in patients requiring life support systems and cannot be performed in patients with implanted electronic devices such as pacemakers, cochlear implants, or defibrillators. A study comparing MR angiography with DSA in renal donors revealed similar accuracy in detecting renal vascular anatomy[12].

### ABNORMALITIES IN DEVELOPMENT OF THE IVC

Obernosterer et al.[13] screened consecutive patients with an anomalous IVC for concomitant abdominal and pelvic arterial abnormalities, and their first-degree relatives for congenital vascular anomalies. MR angiography identified accessory renal arteries in two out of five such patients. Five of the 11 first-degree relatives were found to have major abdominal vascular anomalies including accessory renal arteries, accessory renal veins, and anomalies of the hepatic artery. None of the relatives showed abnormalities of the IVC. The familial occurrence of vascular anomalies strongly suggests an underlying pathogenetic component in affected family members.

Huntington and McLure[2] proposed 14 possible theoretical variations in the anatomy of the IVC. We will be discussing only the following four abnormalities in this article, as the remaining abnormalities of IVC are quite rare in occurrence[2]. Circumcaval ureter, though common, has been excluded as this entity does not require any intervention related to the IVC.

1. Left-sided IVC
2. Double IVC
3. Retroaortic renal vein
4. Circumaortic renal vein

### Left-Sided IVC

Typically, the left IVC joins the left renal vein, which crosses anterior to the aorta in normal fashion uniting with the right renal vein to form normal right-sided, prerenal IVC. It is present in 0.2–0.5% of the human population[7].

The embryological defect with this anomaly is regression of the right supracardinal vein with persistence of the left supracardinal vein or may be seen in patients with situs inversus.

Clinically, the patients are usually asymptomatic. It can be suspected in patients when chest X-rays show dextrocardia. On CT scan of the abdomen, a left-sided IVC can be misdiagnosed as lymphadenopathy on single CT section without contrast. Inspection of the serial sections after contrast administration reveals the tubular nature of the image and thus avoids misdiagnosis as left-sided para-aortic lymphadenopathy[14,15]. The clinical importance of the left IVC is during transjugular access of infrarenal left IVC (for placement of an IVC filter may be difficult) or during left-sided donor nephrectomy.

### Double IVC

Typically, the IVC presents bilaterally and the left renal vein ends into the left IVC, which crosses anterior to the aorta in the normal fashion to join the right IVC. There may be discrepancy in the size of both the IVC. Prevalence of this anomaly is around 0.2–3%[7].

Embryologically, this occurs as a result of the persistence of the caudal portion of the supracardinal vein on the left-hand side.

Usually, this anomaly is asymptomatic and is often diagnosed during retroperitoneal surgery. Clinically, the significance is in patients with deep vein thrombosis who continue to have recurrent pulmonary embolism despite placement of an IVC filter. It can be misdiagnosed as retroperitoneal lymphadenopathy. 3D CT scan with contrast is the diagnostic investigation of choice.

During donor nephrectomy, usually the left-sided kidney is preferred due to the longer length of the renal vein, but the presence of double IVC or left-sided IVC may shorten the length of the renal vein in the graft that may require an IVC cuff. The laparoscopic surgeon should, therefore, mobilize the kidney using laparoscopic armamentarium, but the graft should be harvested by a lumbar incision after applying vascular clamps on the IVC in order to achieve adequate length of the renal vein. However, a skilled laparoscopic surgeon may be able to apply laparoscopic Satinsky clamps and complete the entire procedure. To prevent IVC thrombosis, gentle dissection with minimal retraction is advisable.

Preoperative imaging allows for better planning of the surgical procedure and decreases the morbidity. Duplication of the IVC increases the incidence of vascular complications. Preoperative 3D CT angiography is the preferred mode of evaluation of donors and is being done at our center. It provides rapid and accurate evaluation of the venous system. Preoperative detection enables the planning of the surgical procedure as per the individual case.

### Retroaortic Renal Vein

Embryologically, this results from persistence of the dorsal arch of the renal collar, while the ventral arch of the renal collar regresses, leading to a single renal vein that passes posterior to the aorta. Prevalence of this anomaly is around 2%[7,16]. This is usually asymptomatic, but it has been implicated as a cause of hypertension by nutcracker phenomenon and sometimes it may present as hematuria[17]. Clinical significance of this anomaly is preoperative recognition to avoid intraoperative vascular complications. In donor nephrectomy, the aorta should be gently retracted forward to gain extra length of the renal vein.

We have now acquired much experience with laparoscopic donor nephrectomy[18]. In our initial experience with the transperitoneal approach, we too found it difficult to obtain adequate length as the aorta became a limiting factor and it was not possible to go posterior to the aorta to gain adequate renal vein length except on one occasion when it ran obliquely downward to join the IVC. On at least two occasions, when the course of the vein was straight, we finally resorted to completing the procedure by opening through a lumbar incision and harvesting the kidney. If the laparoscopic approach is adapted, then it is advisable to undertake the retroperitoneal approach where one can safely dissect posterior to the aorta in order to gain extra length of the renal vein[19].

### Circumaortic Renal Vein

This results due to the persistence of the dorsal limb of the embryonic left renal vein and dorsal arch of the renal collar (intersupracardinal anastomosis). Prevalence of this anomaly in a study of 433 abdominal CT scans was 4.4%[20]. There are two renal veins: one passing anterior while another passes posterior to the aorta. The posterior renal vein lies 1–2 cm lower than the anterior renal vein and the former receives the gonadal vein. The major clinical significance of this anomaly is preoperative planning prior to nephrectomy and renal vein sampling. Misdiagnosis as retroperitoneal lymph nodes should be avoided. Presence of a circumaortic left renal vein makes embolization of the symptomatic varicocele technically impossible[21].

In case of circumaortic renal vein, in our experience as well as that of others, the posterior branch of the vein usually has been found to be small and can be controlled with clips, while the larger anterior vein is used for venous anastomosis.

Because of the potential for inadvertent venous injury, the presence of circumaortic and retroaortic left renal vein was considered contraindication for laparoscopic donor nephrectomy. But recently it has been shown that these venous anomalies of the left renal vein do not adversely affect the graft outcome. A left kidney with vasculature anatomically adequate for transplantation can be achieved with excellent donor and recipient outcomes[19].

### REFERENCES

1. Abernethy, J. (1793) Account of two instances of uncommon formation in the viscera of human body. *Philos. Trans. R. Soc*. **83**, 59–66.
2. Huntington, G.S. and McLure, C.F.W. (1920) The development of the veins in the domestic cats with special reference to share taken by supracardinal vein in the development of the post cava and its tributaries, as found in the abould. *Anat. Rec*. **20**, 1–29.
3. Giordano, J.M. and Trout, H.H., 3rd (1986) Anomalies of the inferior vena cava. *J. Vasc. Surg*. **3(6),** 924–928.
4. Zaman, W., Kumar, A., Gupta, R., Das, S., Mandhani, A., Srivastava, A., and Gupta, A. (2002) Helical CT angiography: a single imaging modality to evaluate a live renal donor. *Indian J. Urol*. **18**, 131–135.
5. Kaynan, A.M., Rozenblit, A.M., Figueroa, K.I., Hoffmann, S.D., Cyamon, J., Karwa, G.L., Tellis, V.A., and Lerner, S.E. (1998) Use of spiral computerized tomography in lieu of angiography for preoperative assessment of living renal donors. *J. Urol*. **161**, 1769–1775.
6. Mandal, A.K., Cohen, C., Montgomery, R.A., Kavoussi, L.R., and Ratner, L.E. (2001) Should the indications for laparoscopic live donor nephrectomy of the right kidney be the same as for the open procedure? Anomalous left renal vasculature is not a contraindication to laparoscopic left donor nephrectomy. *Transplantation* **71**, 660–664.
7. Philips, E. (1969) Embryology, normal anatomy and anomalies. In *Venography of the Inferior Vena Cava and its Branches*. Ferris, E.J., Hippona, F.A., Kahn, P.C., Philips, E., and Shapiro, J.H., Eds. Williams and Wilkins, Baltimore. pp. 1–32.
8. Rubin, G.D., Dake, M.D., and Napel, S. (1994) Spiral CT of renal artery stenosis. Comparison of three dimensional rendering techniques. *Radiology* **190**, 181–189.
9. Rubin, G.D., Alfrey, E.J., Dake, M.D., Semba, C.P., Sommer, F.G., Kuo, P.C., et al. (1995) Assessment of living renal donors with CT scan. *Radiology* **195**, 457–462.
10. Platt, J.F., Ellis, J.H., Korobkin, M., and Reige, K.A. (1998) Potential renal donors: comparison of conventional imaging with helical CT imaging. *Radiology* **198,** 419–425.
11. Prince, M.R., Narishaman, D.J., Staney, J.C., Chenevert, T.L., Williams, D.M., Marx, M.V., et al. (1995) Breath hold Gadolinium enhanced MR angiography of abdominal aorta and its major branches. *Radiology* **197**, 785–792.
12. Adams, M.K., Goldszer, R.C., Pulde, M.F., et al. (1995) Renal vasculature in potential renal transplant donors. Comparison of MR imaging and digital subtraction angiography. *Radiology* **197**, 467–472.
13. Obernosterer, A., Aschauer, M., Mitterhammer, H., and Lipp, R.W. (2004) Congenital familial vascular anomalies: a study of patients with an anomalous inferior vena cava, and of their first-degree relatives. *Angiology* **55(1),** 73–77.

14. Siegfried, M.S., Rochester, D., Bernstein, J.R., and Milner, J.W. (1983) Diagnosis of inferior vena cava anomalies by computerized tomography. *Comput. Radiol*. **7**, 119–123.
15. Royal, S.A. and Callen, P.W. (1979) CT evaluation of IVC and renal vein. *AJR Am. J. Roentgenol*. **134**, 759–763.
16. Reed, M.D., Friedman, A.C., and Nealey, P. (1982) Anomalies of the left renal vein: analysis of 433 CT scans. *J. Comput. Assist. Tomogr*. **6(6),** 1124–1126.
17. Gibo, M. and Onitsuka, H. (1998) Retroaortic left renal vein with renal vein hypertension causing hematuria. *Clin. Imaging* **22(6**), 422–424.
18. Kumar, A., Chaudhary, H., Srivastava, A., and Raghavendran, M. (2004) Laparoscopic live-donor nephrectomy: modifications for developing nations. *BJU Int*. **93(9),** 1291–1295.
19. Lin, C.H., Steinberg, A.P., Ramani, A.P., Abreu, S.C., Desai, M.M., Kaouk, J., Goldfarb, D.A., and Gill, I.S. (2004) Laparoscopic live donor nephrectomy in the presence of circumaortic or retroaortic left renal vein. *J. Urol*. **171(1),** 44–46.
20. Monreal, M., Lafoz, E., Casals, A., et al. (1991) Occult cancer in patients with deep venous thrombosis. *Cancer* **67,** 541–545.
21. Aguilar Rivilla, E., de la Pena Zarzuelo, E., Gallego Beuter, J., Mendez Montero, J.V., Santos Martin, E., Delgado Martin, J.A., and Blazquez Izquierdo, J. (2002) Difficulties in the treatment of symptomatic varicocele using percutaneous embolization: the circumaortic renal vein. *Arch. Esp. Urol*. **55(5),** 497–500.

**This article should be referenced as follows:**

Srivastava, A., Singh, K.J., Suri, A., Vijjan, V., and Dubey, D. (2005) Inferior vena cava in urology: importance of developmental abnormalities in clinical practice. *TheScientificWorldJOURNAL* **5**, 558–563.

**Handling Editor:**

A. Atala, Principal Editor for *Urology* and *Cell Biology* — domains of *TheScientificWorldJOURNAL*.
